# A Scale to Measure the Joy in Work of Doctors: Development, Validity, and Reliability

**DOI:** 10.3389/fpubh.2021.760647

**Published:** 2021-12-20

**Authors:** Zemiao Zhang, Yinhuan Hu, Hao Chen, Weilin Zhu, Dehe Li, Ximin Zhu, Xiaoyue Wu, Jiayi Li

**Affiliations:** School of Medicine and Health Management, Tongji Medical College, Huazhong University of Science and Technology, Wuhan, China

**Keywords:** doctors, joy in work, scale, reliability, validity

## Abstract

**Background:** The aim of this study is to develop a scale and evaluate its' validity and reliability to measure the joy in work of doctors.

**Methods:** Based on literature review and panel discussion, the scale framework and item pool were determined. Next, the items were modified by two rounds of expert consultation. Then the pre-investigation was applied and the formal version of scale was formed. Last, the reliability and validity of the scale were tested with 426 physicians.

**Results:** The scale was composed of four dimensions: work autonomy needs, competency identification needs, competency perception needs and work relationship needs. Each dimension had 7 items, and both reliability and validity were acceptable. The Cronbach α coefficient and half-reliability coefficient of the whole scale were 0.954 (>0.9) and 0.974 (>0.9). The Spearman correlations of item-total score ranged from 0.556 to 0.749, indicating a good-item total score correlation. The χ 2/ df, RMSEA, RMR, GFI, CFI, and TLI, CFA of the maximum likelihood method supported a good fit with the model.

**Conclusions:** Based on the self-determination theory, this study develops a scale to measure the joy in work of doctors. It has good validation and reliability, which is useful for doctors and medical institutions to take steps to improve happiness.

## Introduction

The mental health of healthcare workers has come under renewed attention during the COVID-19 pandemic. The proportion of doctors who felt panic, helplessness, loneliness, fatigue, mental distress, anxiety and depression increased significantly due to the negative impact of work stress and patient outcomes (1–3). A research in Wuhan showed that the percentage of depression, anxiety, insomnia and pain of front-line medical staff were 50.4, 44.6, 34.0, and 71.5%, respectively (2). A systematic review of 13 studies from the UK revealed that the prevalence of anxiety, depression, and insomnia was 23.2, 22.8, and 38.9%, respectively (4). In this case, the mental health of medical staff has become a priority in the world. Three ministries in China (National Health Commission, Ministry of Human Resources and Social Security, Ministry of Finance) jointly issued a document pointing out that “Psychological crisis intervention should be strengthened to relieve the psychological pressure of medical personnel” (5).

In fact, the psychological problems of clinicians have been paid attention for a long time. With the rapid development of medical technology and aggravation of work challenges, doctors tend to have low job satisfaction (6, 7). A meta-analysis of 9,302 doctors showed that the prevalence of professional burnout among Chinese doctors ranged from 66.5 to 87.8%, especially for those aged 30–40 years old (8). Another survey found that staff turnover among physicians has increased sharply in recent years, with 60% considering leaving (9). According to 2018 British Medical Association online survey, as doctors work longer hours, they are more likely to suffer from emotional disorders (10). A large number of studies have shown that stressful working conditions of doctors not only adversely affect individual health, but also endanger patient safety and medical quality (11).

In this case, the Institute for Healthcare Improvement (IHI) and Johnson Foundation proposed that we should think about how to improve fun at work rather than how to solve mental problems (12). In their view, concentrating on negative emotions can cause people to magnify problems rather than solve them. Slaughter first defined “fun” in the field of psychology as an expression of individual quality of life that can be achieved through intellectual activity, behavioral activity, and emotional experience (13, 14). Lamm insisted that work fun is social, interpersonal and entertaining (15). In addition, work pleasure is often related to efficiency and achievement (16, 17). By focusing on the joy of the physician's work, it inspires key resources such as care, compassion, and dedication that benefit relationships and organizational culture (18, 19).

However, there is no effective instrument for measuring doctors' pleasure at work. Development of work fun scale is limited to enterprise management. Ford et al. developed an instrument covering ten kinds of activities in 2003 (19). Karl and Peluchette established a scale including 40 items (20). Through focus group discussion, expert consultation, exploratory and confirmatory factor analysis, McDowell constructed the scale from four dimensions: socializing with coworkers, celebration at work, personal freedoms and global fun, with six items in each dimension (21). Chan et al. summarized a useable typology “4S” model of work fun in the hospitality industry, but only ten practitioners participated in the survey (22). From the perspective of brand building, Liang Yuqing proposed that work fun includes customer interaction, coworker relationships and enjoy oneself. The Cronbach α coefficient of scale was 0.976 and construct reliability (CR) was 0.993 (23). Wang et al. revised the Chinese Workplace Fun Scale. Data analysis showed that the scale has good reliability and validity. They also said that different types of workplace fun have different effects on employees' performance. But this argument still requires more research to verify in the future (24). To sum up, most of scales of work pleasure are applicable to enterprise management, and many of them only stay in theoretical construction stage, lacking empirical test. Different from corporate employees, doctors have strict work regulations. What is more, the existing scale lacks a reflection of the pleasure of work itself. Some of the latest pleasures are not included. So far, work evaluations of clinicians still use old tools such as working pressure scale, job burnout scale, turnover intention, organizational atmosphere, etc. Happiness is a positive experience in work, and it is also the life goal that people pursue. To enrich the work evaluation of doctors, this paper tries to develop a scale to measure the joy in work of doctors and test its reliability and validity using mixed methods. It is hoped that this study can better understand the working conditions of doctors and provide reference for doctors and medical institutions to take corresponding measures to improve happiness.

## Methods

### Study Design

**Figure 1:**
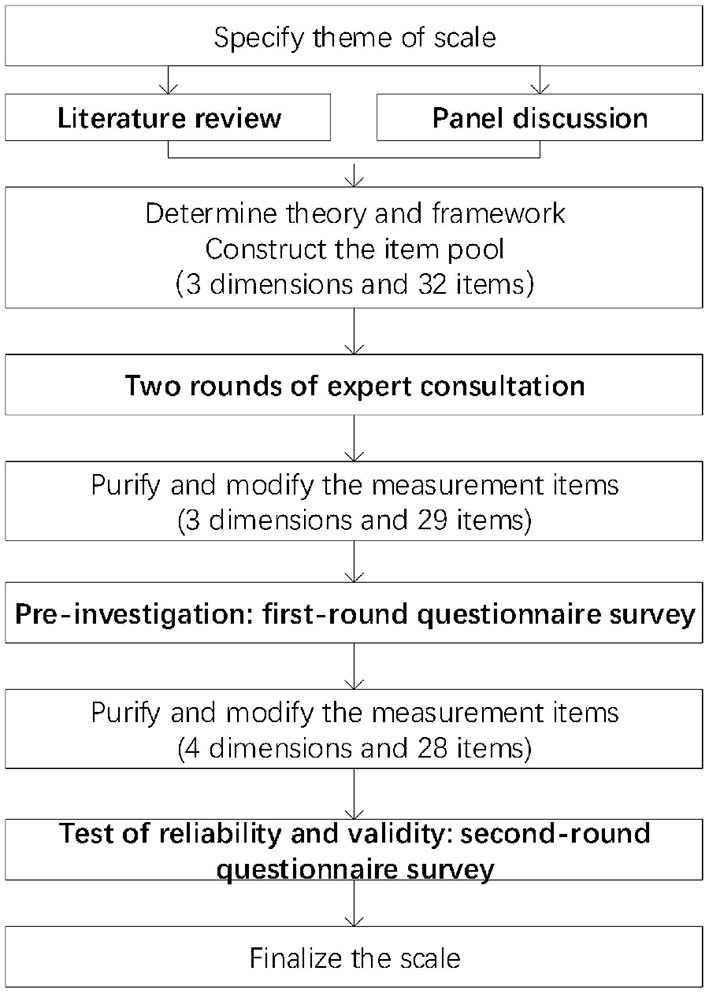
Procedure for scale development.

The scale was developed in four phases. First, we determined the basic theory and constructed the item pool through extensive literature search and panel discussion. Second, the generated items were reviewed by two rounds of expert consultation. Third, we conducted the pilot survey and modified items and dimensions. Forth, we carried out a formal questionnaire survey and assessed reliability and validity of the scale (see Figure 1).

### Phase 1: Building the Entry Pool and Determining the Scale Structure

On Google academic, Web of science, Elsevier, CNKI and other websites, we took “fun at work” “fun in work” “joy in work” “workplace fun” and “doctor” “physician” “medical staff” as key words to collect the related resource. Many scales were referenced to build the entry pool, such as Liang's work pleasure scale, Minnesota Satisfaction Questionnaire, Job satisfaction scale, McDowell's work pleasure scale, Niehoff and Moorman's fairness scale, Wang Yaming scale, Tews workplace pleasure scale, etc. (19–24). After comparing many theories, we decided to build the scale framework based on self-determination theory. According to the theory, individual happiness and active behavior are caused by the satisfaction of three psychological needs: the need for competence, the need for autonomy and the need for relationship. All items were expressed in a positive way. That is, if the actual situation is consistent with the item description, the doctor's work fun will be high. Back translation was used to ensure the validity of all English sentences. Through three rounds of panel discussion, the uncertain index items were defined and analyzed. Finally, three dimensions and 32 items were determined.

### Phase 2: Identifying the Items by Expert Consultation

The expert consultation form was developed based on the preliminary item pool. The form included experts' information, judgment basis and the importance of each item. The initial scale was sent to experts by email or in person and they were asked to give feedback within the agreed time. A total of 20 experts were invited. After each round of consultation, items were edited according to the critical value of mean, coefficient of variation and full score ratio. After two rounds of consultation, experts' opinions tend to be consistent, indicating that dimension and item selection and cross-cultural Chinese language adaptation have been completed (25). In accordance with the findings from expert consultation, we deleted three items and revised the descriptions of seven items. The dimension of autonomy needs, competency needs and relationship needs contains six items, 15 items and eight items, respectively, with a total of 29 measurement items. The characteristics of experts and details of consultation can be seen in our another article.

### Phase 3: Conducting the Pre-investigation

The 29 generated items were piloted among 226 doctors by random sampling and snowball sampling. Appendix 1 gave the characteristics of doctors. To ensure the representativeness, sensitivity, importance and independence of items, frequency distribution analysis, critical ratio method, variation coefficient (VC) and Cronbach α Coefficient, correlation analysis and exploratory factor analysis (EFA) were adopted to filter and correct items (26, 27). Appendix 2 provided a detailed operation process. Considering the above six methods, if an item was excluded by two or more methods, the item would be revised or deleted from the scale. The result (KMO = 0.944, Bartlett's test value = 4898.860, *P* < 0.001) indicated perfect appropriateness to conduct exploratory factor analysis. According to the results of factor analysis, the dimension of competency needs was divided into competency perceived needs and competency identified needs. The former meant that something itself can bring pleasure to the individual. The latter referred to something that brings pleasure to the individual from an external environment or related connection. According to the calculation results, we deleted one item and modified 1 item in this phase. The adjusted scale had four dimensions and 28 items.

### Phase 4: Testing the Reliability and Validity of Scale

#### Participants

The formal survey was conducted in March and April 2021. Our participants were doctors working in medical institutions. The formal survey scale consisted of two parts. The scale instructions indicated that our scale was anonymous, participants were voluntary, and our survey aimed to develop a scale to measure their happiness at work. The first part included 28 items that respondents scored one by one. Each item was designed with 5-level Likert scale, and the options were “extremely disagree,” “disagree,” “general,” “agree,” and “extremely agree.” The second part was designed to collect respondents' individual characteristics.

Considering the sample size recommended for factor analysis, we decided that the number of physicians surveyed should be 10 times the number of projects (27). At first, we chose a local hospital for investigation, which is a tertiary comprehensive public hospital in Hubei province, as well as a university hospital. Seventy questionnaires were distributed with assistance from leaders and the department of medical service. The doctors gave positive cooperation and all questionnaires were recovered. Due to the COVID-19, we also adopted the form of online survey using the survey website wenjuanxing (www.wjx.cn) to collect the view of doctors in other areas. Respondents could scan the access code or click on the website using their computers or phones to access and complete the electronic questionnaire. We sent the access code and website to the human resource managers in hospitals, who then sent the access code to the doctors' online communication groups in their hospitals and WeChat circles of friends. To prevent data duplication, each IP address was allowed to fill in questionnaire only once. We connected nine leaders of hospitals situated in different provinces and 356 doctors voluntarily participated in this survey. Finally, a total of 426 questionnaires were obtained, which met the requirements of sample size.

Ethics approval was obtained from the Ethics Committee of Tongji Medical College, Huazhong University of Science and Technology. All the data were kept confidential and anonymous. Doctors who participated in the offline survey can get a vial of hand sanitizer. Doctors who submitted the online questionnaire will enter a lucky draw and receive an average of 1.5 yuan in red envelopes. Only fully completed questionnaires were valid for this study.

### Statistical Analysis

The EpiData entry 3.1 software (the EpiData Association, Odense, Denmark) was used to establish and manage the database. To ensure the accuracy of data, double-entry pattern was adopted. The score of scale was the sum of scores of each item. Statistical analysis was performed using IBM SPSS statistics 22.0 software (IBM, Armonk, NY) and IBM SPSS Amos 17.0 software (IBM, Armonk, NY).

The reliability and validity of 28 items were tested. Reliability referred to the consistency and stability of test results and was examined by Cronbach α Coefficient and split-half reliability coefficient. Validity referred that the instrument can measure the degree of object, including content validity and construct validity. The former was verified with content validity index (CVI). The latter was tested by correlation analysis and confirmatory factor analysis.

### Reliability

When calculating split-half reliability coefficient, we divided the items into an odd group and even groups according to the serial number. Then the correlation coefficient between the two groups was calculated, and the Spearman-Brown formula was applied to estimate the reliability of the whole scale. It is agreed that the split-half reliability coefficient should be >0.7 to reach a reasonable level. The Cronbach α coefficients of the whole scale and each dimension were calculated to evaluate the internal consistency of the scale. The value ranges from 0 to 1. The larger the value, the better the consistency. Generally, values above 0.7 were considered acceptable results (28).

### Content Validity

Content validity of the scale was measured by CVI method and item relevance scoring method based on expert consultation. Experts were invited to rate items on a scale of 1–4 (1 being irrelevant, 2 being relevant, 3 being fairly relevant, and 4 being highly relevant) on their relevance to the dimensions they belong to. Project-level CVI (I-CVI) was the number of experts giving a 3 or 4 point evaluation for each project divided by the number of experts and is generally expected to exceed 0.78. Considering the randomness of experts' scoring, we used formula ${\text{P}}_{\text{C}}=\left[\frac{n!}{A!(n-A)!}\right]\;\times \;0.5^{n}$ to obtain the corrected I-CVI. If the corrected I-CVI were >0.6, it could be admitted as an acceptable level. If the corrected I-CVI were >0.74, it could be considered as an excellent degree. For scale-level CVI (S-CVI), the scale-level content validity/universal agreement (S-CVI/UA) should be >0.8, and the scale-level content validity index/average (S-CVI/AVE) should be >0.9 to reach an ideal level.

### Construct Validity

Correlation analysis and confirmatory factor analysis (CFA) were used to test the validity of the structure. Correlation analysis includes three parts: item-total score correlation coefficient, dimensional-total score correlation coefficient and dimensional-dimension correlation coefficient. The reasonable range was (0.3, 0.8), (0.3, 1), (0, 0.8), respectively (29). Only when all coefficients meet the conditions can it be proved that the scale has good relevance and discrimination. Bartlett test of sphericity scores <0.05 and a KMO score of sampling degree >0.70 and close to 1 were considered appropriate for factor analysis (30). As for CFA, model fit indications such as χ^2^/df, root mean square error of approximation (RMSEA), comparative fit index (CFI), goodness-of-fit index (GFI), Tucker-Lewis index (TLI), and root mean square residual (RMR) were used to evaluate the model fit. In general, if χ^2^/df < 3, RMSEA < 0.08, CFI > 0.90, GFI > 0.90, TLI > 0.90, and RMR < 0.09, indicating that the goodness-of-fit index is reasonable and acceptable (31).

## Results

### Respondent Characteristics

Four hundred and twenty six questionnaires were received (70 offline and 356 online) and all were valid, with an effective recovery rate of 100%. The number of doctors from Hubei, Guangxi, Shanxi, Guangdong, Beijing were 70, 56, 56, 42, 40, respectively, accounting for most of the respondents. Other areas were Shandong, Jiangxi, Shanghai, Hebei, Jiangsu. Among them, 154 (36.15%) were male and 272 (63.85%) were female. Most of them were under 45 years old (392, 92.02%). The number of resident and attending doctors were 236 (55.40%) and 134 (31.45%), accounting the most of sample. In terms of educational background, the respondents were mainly undergraduates (287, 67.37%) and masters (96, 22.54%). The number of doctors from tertiary hospital, secondary hospital and primary hospital were 226 (53.05%), 161 (37.80%), and 39 (9.15%), respectively. Respondents characteristics are shown in Table 1.

**Table 1:** Demographic characteristics of respondents.

**Variables**	**Category**	***N*** **(%)**	**Variables**	**Category**	***N*** **(%)**
Age (years)	≤24	37 (8.69)	Working years	1–5	227 (53.28)
	25–34	185 (43.43)		6–10	100 (23.47)
	35–44	109 (25.59)		11–15	45 (10.56)
	45–54	59 (13.85)		≥16	54 (12.68)
	≥55	36 (8.45)	Department	Internal	116 (27.23)
Gender	Male	154 (36.15)		Surgery	96 (22.54)
	Female	272 (63.85)		Obstetrics	46 (10.80)
Title^*^	Resident	236 (55.40)		Gynecology	31 (7.28)
	Attending	134 (31.45)		Others	137 (32.16)
	Deputy chief	48 (11.27)	Hospital level	Primary	39 (9.15)
	chief	8 (1.88)		Secondary	161 (37.80)
Educational degree	Diploma or below	20 (4.69)		Tertiary	226 (53.05)
	Bachelor's	287 (67.37)			
	Master's	96 (22.54)			
	PhD	23 (5.4)			

* *Different from other countries, the rank of titles of doctors in China has four types: resident doctor, attending doctor, deputy chief doctor, and chief doctor*.

### Reliability

#### Split-Half Reliability Coefficient

The 28 items were divided into odd group (A1, A3, A5, A7, B2, B4, B6, C1, C3, C5, C7, D2, D4, D6) and even group (A2, A4, A6, B1, B3, B5, B7, C2, C4, C6, D1, D3, D5, D7). Statistical analysis showed that the correlation coefficient between two groups was 0.949 and the split half reliability coefficient was 0.974, which met the standard above 0.7, indicating that the scale has a good internal reliability.

#### Cronbach α Coefficient

After calculation, the overall Cronbach α coefficient value was 0.954, which meet the standard requirements (>0.7), indicating that the structure of scale was reasonable. The value of Cronbach α coefficient of four dimensions (independent needs, competency identification needs, competency perception needs and relationship needs) were 0.872, 0.864, 0.892, and 0.912, respectively, which were >0.70, indicating that the scale has good internal consistency.

### Content Validity

The experts we invited were the same as the experts in second round consultation. As can be seen from the corrected I-CVI in Table 2, all items has reached a good level except for B6 and C3. But they were all >0.6, which was considered acceptable. For S-CVI, the S-CVI/UA was 0.571 and the S-CVI/Ave was 0.92 (>0.9). Although S-CVI /UA was lower than 0.8, research showed that the value of S-CVI/UA would decrease as the number of specialists increased. Therefore, the true overall content validity of the scale may be between S-CVI/UA and S-CVI/Ave, which can be considered an acceptable level.

**Table 2:** Corrected I-CVI of items.

**Dimensions/Items**	**I-CVI**	**Corrected I-CVI**
**A. Work autonomy needs**		
A1. I have some autonomy in my work schedule	1	1
A2. I think my workload is appropriate	1	1
A3. I have the flexibility to decide how and how to accomplish tasks	0.93	0.92
A4. I can make work decisions based on my own judgment	1	1
A5. I can reasonably express emotions or opinions in the workplace	1	1
A6. I can have a proper rest during my work	0.83	0.82
A7. I am very focused in my work and I don't get interrupted	1	1
**B. Competency identification needs**		
B1. I think my work is of great significance	1	1
B2. I think my work is creative	0.80	0.79
B3. I think my job is stable	0.80	0.76
B4. I have learned and improved in my work	1	1
B5. I meet new things and have different experiences in my work	1	1
B6. My abilities are recognized by my colleagues and it's a pleasure to work with them	0.75	0.72
B7. My colleagues and I help each other to complete tasks and feel a sense of belonging	1	1
**C. Competency perception needs**		
C1. I think my work itself is respected	0.8	0.79
C2. I can give full play to my ability in work, meet the needs of patients, and feel happy/satisfied	0.8	0.79
C3. I can communicate happily with patients (relatives) at work, which is helpful for disease treatment	0.67	0.64
C4. I have made outstanding achievements in my work and feel a sense of achievement	1	1
C5. I have completed challenging tasks and experienced the joy of overcoming difficulties	1	1
C6. I feel comfortable with the space where I can give full play to my expertise in my work	0.93	0.92
C7. I have the opportunity to play an important role in the team and achieve excellence	1	1
**D. Work relationship needs**		
D1. I am satisfied with the commendation activities of the hospital (such as commendation of excellent employees, award of outstanding achievements, etc.)	0.87	0.86
D2. I am satisfied with the welfare provided by the hospital after work (such as issuing daily necessities, food, shopping cards, holding lucky draw, product auction, new year's party, etc.)	0.93	0.92
D3. I am satisfied with the activities organized by the Department for employees' personal or family (such as personal birthday, entry anniversary, visiting family members on holidays or mailing gifts, etc.)	1	1
D4. My superiors treat people equally	1	1
D5. My superior is trustworthy and gives guidance to my work. We have a harmonious relationship	1	1
D6. My organization is fair, and my job can be fairly rewarded	0.93	0.92
D7. Equal opportunities for promotion. I am satisfied with the promotion mechanism of the hospital	1	1

### Construct Validity

#### Correlation Analysis

As shown in Table 3, A, B, C, D and T were significantly correlated at α = 0.01 level, and the Spearman correlation coefficient ranged from 0.617 to 0.886, showing a positive correlation. The correlation coefficients between dimensions were 0.617–0.785, all <0.8. The correlation coefficients of dimension-total scores were >0.8, reaching an optimal level. The correlation analysis between items and total scores were shown in Table 4. The Spearman correlations of item-total score ranged from 0.556 to 0.749, indicating a good-item total score correlation.

**Table 3:** Spearman correlations of inter-dimension and dimension-total score.

**Dimensions**	**A**	**B**	**C**	**D**	**T**
A	1				
B	0.617^**^	1			
C	0.625^**^	0.785^**^	1		
D	0.705^**^	0.668^**^	0.655^**^	1	
T	0.858^**^	0.865^**^	0.867^**^	0.886^**^	1

** *The correlation is remarkable at α = 0.01 (double end)*.

**Table 4:** Spearman correlations of items and corresponding dimensions or total score.

**Items**	**Correlation between items and corresponding dimensions**	**Correlation between items and total scores**	**Items**	**Correlation between items and corresponding dimensions**	**Correlation between items and total scores**
A1	0.798^**^	0.663^**^	C1	0.727^**^	0.654^**^
A2	0.767^**^	0.648^**^	C2	0.794^**^	0.679^**^
A3	0.777^**^	0.609^**^	C3	0.777^**^	0.638^**^
A4	0.729^**^	0.619^**^	C4	0.815^**^	0.660^**^
A5	0.772^**^	0.662^**^	C5	0.778^**^	0.624^**^
A6	0.748^**^	0.648^**^	C6	0.816^**^	0.749^**^
A7	0.678^**^	0.677^**^	C7	0.757^**^	0.726^**^
B1	0.773^**^	0.612^**^	D1	0.822^**^	0.743^**^
B2	0.782^**^	0.689^**^	D2	0.816^**^	0.681^**^
B3	0.635^**^	0.556^**^	D3	0.820^**^	0.684^**^
B4	0.804^**^	0.697^**^	D4	0.800^**^	0.689^**^
B5	0.767^**^	0.702^**^	D5	0.794^**^	0.711^**^
B6	0.664^**^	0.584^**^	D6	0.809^**^	0.774^**^
B7	0.775^**^	0.654^**^	D7	0.811^**^	0.748^**^

** *The correlation is remarkable at α = 0.01 (double end)*.

#### Confirmatory Factor Analysis

The results (KMO = 0.955, Bartlett's test value = 7368.996, *P* < 0.001) indicated appropriateness to conduct confirmatory factor analysis. For CFA, the model fit indices were χ^2^/df = 1.450 (<3.000), RMSEA = 0.033 (<0.08), GFI = 0.920 (>0.90), CFI = 0.908 (>0.90), TLI = 0.907 (>0.90), and RMR = 0.052 (<0.09). From these indicators, it can be seen that the construct validity of the scale has reached an acceptable level.

## Discussion

It is necessary to solve the doctor's job burnout and bad emotions from the perspective of positive psychology. However, there is still a lack of available public standards to evaluate the doctors' work joy. Based on the theory of self-determination, this study attempts to develop an instrument to evaluate the doctors' work pleasure by qualitative and quantitative methods. According to the test results, this scale has performed well in the acceptability, validity and reliability among Chinese doctors. The formal scale consists of 28 items, covering four factors: work autonomy needs, competency identification needs, competency perception needs and work relationship needs.

The first dimension, work autonomy needs, refers to the ability of individuals to decide their own behavior based on their real will when faced with choices. When this need is met, doctors will experience the flexibility and freedom of work, and then get a sense of happiness (32). According to the literature and data analysis, this factor was measured from seven aspects: work time, workload, methods, decision-making, work-life balance, emotion and attention. After expert consultation, it is quite reasonable to exclude the item “I can do what I like to do at work,” because it does not reach full score ratio. Unlike company employees, doctors' work is serious and institutionalized since human lives are at stake. Different scales are suitable for people of different occupations. Our research also confirms this point. Some people think that “the pace of modern life is too fast, and it needs to sacrifice rest time to complete the work.” In fact, this view is very one-sided and can easily lead to fatigue and depression. Empirical studies show that enhancing employees' autonomy can not only contribute to improve their positive attitude and performance (33), but also increase employees' life satisfaction (34), thus affecting more efficient organizations in turn (35).

According to the results of exploratory factor analysis, competency needs were divided into competency perception needs and identification needs. The item “B1 I think my work is of great significance” indicated that perceiving the meaning of work would bring a happy experience. The work feature model holds that work meaning is an important work feature, which is determined by skill diversity, task integrity and task importance (36). Sikson Mihai, the founder of flow theory, believed that successful people would deeply feel the significance of their work (37). A survey of doctors in Shandong Province showed that the average index of doctors' work significance was 13.24 ± 0.32, which was positively correlated with doctors' income. The lack of work meaning often leads to negative emotional experience (38). In our study, experts agreed that stability was an important indicator of doctors' emotions. Since the new medical reform, doctors' job stability has improved and their intention to leave has weakened (39). However, according to a survey conducted by the Chinese Medical Association in 2017, 64.48% of medical staff did not want their children to go to medical-related profession (40). How to improve the organizational commitment of clinicians is a problem worth studying in the future. In the dimension perceived needs, item C1 stresses that doctors' work is of great social significance and should be respected by the whole society. However, the current situation is not ideal. Even during the epidemic COVID-19, there are still many violent medical injuries. Related studies have identified that violent medical incidents seriously affect job satisfaction and subjective well-being. As caregivers of patients, some doctors complain that their bad emotions are caused by patients, which is not the case. In fact, this kind of cognition is wrong. They would better learn how to find happiness in their interactions with patients (41). Modern medicine advocates patients to actively participate in medical decision-making. Effective communication with patients at work undoubtedly improves doctors' job satisfaction. Items C4C5C6C7 indicates the uncertainty of medical risks and the challenging tasks of the doctors. Advanced hardware facilities are important, but excellent medical technology is the most valuable wealth that brings doctors a sense of professional accomplishment (42).

Previous research have confirmed that relationship needs are very important for happiness at work. In our research, relationship needs is also an important dimension. It has the characteristics of reciprocity, involving not only the connection and association at the individual level, but also the sense of tolerance and harmony at the group level. In the preliminary investigation, we deleted an item based on the Variation Coefficient as well as Cronbach α Coefficient. The relationship dimension is measured from hospital, department, superior and colleagues. The item D1 focuses on the evaluation of commendation and excellence evaluation activities. The items D2 and D3 focus on the evaluation of entertainment activities. Creating a good working atmosphere through celebrations can significantly improve employees' organizational self-esteem, enhance their relationship energy, and thus effectively enhance employees' sense of belonging and meet their relationship needs (28). Most doctors in China are unwilling to communicate with their superiors and dare not express their thoughts on work easily. They often obey unconditionally before authority, which is mainly related to traditional ideas. However, it is such kind of forbearing and introverted character that will easily leads to excessive work pressure and psychological problems. Some researches proposed that by showing altruistic care and help, leaders could not only protect employees from adverse emotions, but also promote their work enthusiasm and initiative behaviors (43).

During the formal investigation, we collected 426 questionnaires (70 online and 369 offline), all of which were valid, and the effective recovery rate was 100%. It was indicated that the scale was highly recognized. Four factors were extracted from factor analysis, which explained 61.697% of the variance. The Cronbach α coefficient and half-reliability coefficient of the whole scale were 0.954 (>0.7) and 0.974 (>0.7), respectively, which indicated that the scale had good reliability. Additionally, after correction, the I-CVI was higher than 0.60, and the S-CVI/AVE was more than 0.9, which showed that it had good content validity. In terms of construct validity, the correlation coefficient between items and total scores was >0.4, indicating that construct validity was good. In addition, as shown by χ^2^/df, RMSEA, RMR, GFI, CFI, and TLI, CFA of the maximum likelihood method supports a good fit with the model.

According to the self-determination theory, if the organizational environment can meet the basic psychological needs of individuals, the internal potential of employees will be activated (44). Positive emotions are directly related to a series of performance behaviors (45). A large amount of evidences show that management practice of focusing on cultivating happy employees can reduce medical errors and improve patient experience. Sharing your happiness with others, and yours will be doubled (46). Zhang Yanling, president of the Chinese Medical Association, pointed out that if one wanted to become a good doctor, he/she should love the profession of doctors, patients, and professional fields, which was the source of happy work (47).

At present, the measurement and evaluation of work pleasure mainly concentrates on the field of enterprises. To our best knowledge, this study was the first to develop a scale to measure the joy in work of doctors in the world based on self-determination theory. The research method includes expert consultation, group discussion and various statistical methods. The research method is scientific, and the scale has high reliability and validity. The scale can be used in practice to evaluate the doctor's happy working degree; It can also be used as a guide for doctors and medical institutions to take corresponding measures to improve the happiness index. However, this study also has some limitations that need attention.

To ensure the representativeness of the samples, we collected opinions of doctors from 10 provinces. Due to the limitations of COVID-19 epidemic, we mainly adopted the form of online questionnaires and the simple size may be small. Most of the groups using the Internet were young and middle-aged doctors, and there might be bias in sampling. It might lead to some problems in generalizability, especially for old doctors. Further research on a wider population is required. Secondly, the classical measurement theory was used to screen the questionnaire items, and the parameters were estimated through sample analysis. There might be errors in the combination, omission and modification of scale items. Thirdly, we cannot deny the limitations of self-reported data as this might limit the validity of the scale. For various reasons, respondents may underestimate or overestimate the experience of their work. It is necessary to research and develop more measuring tools about job pleasure in the future.

## Conclusions

Based on the self-determination theory, this study develops a scale to measure the joy in work of doctors. The scale has four dimensions, each with seven items, and has good reliability and validity. It is an effective tool to study the doctors' work pleasure. It also helps us to find out the factors that affect doctors' happy work, take targeted improvement measures and create an atmosphere of respecting doctors.

## Data Availability Statement

The datasets used and/or analyzed during the current study are available from the corresponding author on reasonable request. Requests to access these datasets should be directed to hyh288@hotmail.com.

## Ethics Statement

Ethics approval was obtained from the Ethics Committee of Tongji Medical College, Huazhong University of Science and Technology (No. IORG0003571). All the survey were kept confidential and anonymous.

## Author Contributions

ZZ conducted the literature review, took part in the investigation, performed formal analysis, and wrote the original draft. YH designed the study, obtained funding, and performed revisions of the manuscript. HC performed formal analysis and performed revisions of the manuscript. WZ conducted the literature review, took part in the investigation, and were involved in data cleaning. DL and XZ were involved in data cleaning and contributed to the interpretation of the results. XW and JL took part in the investigation and contributed to the data cleaning. All authors contributed to the article and approved the submitted version.

## Funding

This work was supported by the National Natural Science Foundation of China (grant number 71774062). Funder was not involved in the design, delivery, or submission of the research.

## Conflict of Interest

The authors declare that the research was conducted in the absence of any commercial or financial relationships that could be construed as a potential conflict of interest.

## Publisher's Note

All claims expressed in this article are solely those of the authors and do not necessarily represent those of their affiliated organizations, or those of the publisher, the editors and the reviewers. Any product that may be evaluated in this article, or claim that may be made by its manufacturer, is not guaranteed or endorsed by the publisher.

## APPENDIX

**Appendix 1:** Demographic characteristics of respondents in pilot survey (*N* = 226).

**Variables**	**Category**	***N*** **(%)**	**Variables**	**Category**	***N*** **(%)**
Age (years)	≤24	26 (11.50)	Working years	1–5	111 (49.12)
	25–34	100 (44.25)		6–10	51 (22.57)
	35–44	55 (24.34)		11–15	30 (13.27)
	45–54	19 (8.41)		≥16	34 (15.04)
	≥55	26 (11.50)	Department	Internal	65 (28.76)
Gender	Male	79 (34.96)		Surgery	49 (21.68)
	Female	147 (65.04)		Obstetrics	29 (12.83)
Educational degree	Diploma or below	20 (8.85)		Gynecology	16 (7.08)
	Bachelor's	141 (62.39)		Others	67 (29.65)
	Master's	59 (26.11)	Hospital level	Primary	16 (7.08)
	PhD	9 (3.98)		Secondary	76 (33.63)
Title	Resident	114 (50.44)		Tertiary	134 (59.29)
	Attending	75 (33.19)			
	Deputy chief	32 (14.16)			
	Chief	5 (2.21)			

**Appendix 2:** Process of editing items in pilot survey.

**Method**	**Operation/ judgment standard**
(1) Frequency distribution analysis	If the choice rate of one item was >85% or <15%, it may have a ceiling or floor effect and should be deleted. Otherwise, it should be retained.
(2) Critical ratio method	We took out the front and rear 27% of the scale and divided them into 2 groups. Then *t*-test was used to identify the difference of each item for the 2 groups. If the *P*-value was >0.05, the item could not identify different subjects and should be deleted. Otherwise, it should be retained.
(3) Variation coefficient method	The reference standard value was 0.15. If it was ≥0.15, it should be retained. Otherwise, it should be modified or deleted.
(4) Cronbach α Coefficient method	First, the Cronbach α Coefficient of the whole scale was calculated. Then an item was deleted. The Cronbach α Coefficient of the residual scale with left items was calculated again. If the Cronbachα Coefficient of the residual scale was higher than the whole scale, the item should be deleted. Otherwise, it should be retained.
(5) Correlation analysis	If the correlation coefficient of an item to its dimension exceeded 0.6, it was representative and should be retained. If the correlation coefficient of an item to other dimensions was <0.5, it had strong independence and should be retained. Otherwise, it should be deleted.
(6) Exploratory factor analysis	The result (KMO = 0.944, Bartlett significance = *P* < 0.000) indicated perfect appropriateness to conduct exploratory factor analysis. If the factor loading of the item was <0.4, the item should be deleted. Otherwise, it should be retained.
